# Heart in An Eggshell Calcification: Idiopathic Calcific Constrictive Pericarditis

**DOI:** 10.4021/cr125w

**Published:** 2011-11-20

**Authors:** Bong Gun Song, Gu Hyun Kang, Yong Hwan Park, Woo Jung Chun, Ju Hyeon Oh

**Affiliations:** a Division of Cardiology, Cardiac and Vascular Center, Department of Medicine, Samsung Changwon Hospital, Sungkyunkwan University School of Medicine, Changwon, Korea

**Keywords:** Constrictive pericarditis, Echocardiography, Computed tomography

## Abstract

Constrictive pericarditis is caused by fibrosis and calcification of the pericardium, which inhibits diastolic filling of the heart. Chest roentgenogram can show the calcification as a mass or sheet over the heart and computed tomography scan allows anatomic delineation of the pericardium and determines the extent of calcification. We reported a case of eggshell calcification of idiopathic chronic constrictive pericarditis diagnosed by echocardiography and multi-detector computed tomography.

## Introduction

Constrictive pericarditis (CP) is caused by fibrosis and calcification of the pericardium, which inhibits diastolic filling of the heart. Careful examination of chest roentgenogram may raise suspicion of calcific CP [1-4]. Computed tomography (CT) scan allows a nice anatomic delineation of the pericardium and determines the extent of calcification by comparing its morphology and density [5-6]. We reported an interesting case of heavy eggshell calcification of idiopathic chronic CP diagnosed by echocardiography and multi-detector CT.

## Case Report

**Figure 1:**
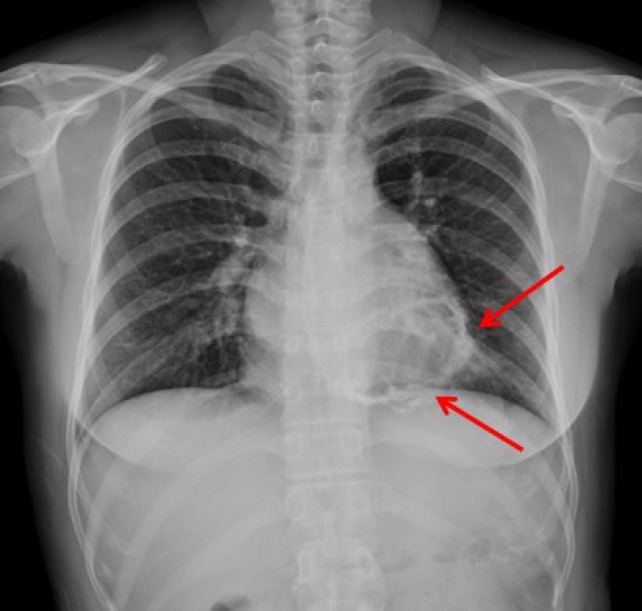
Chest roentgenogram showed normal cardiac size with calcification of the pericardium (arrows).

**Figure 2:**
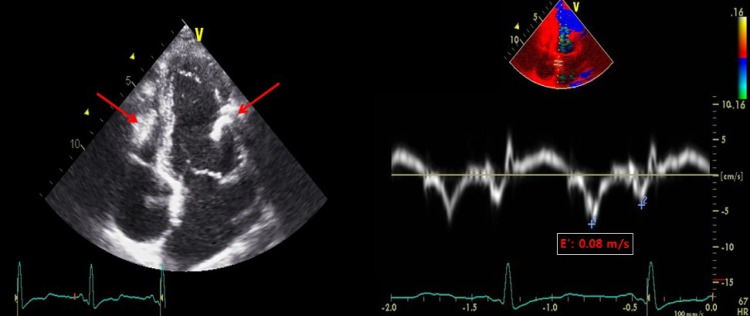
TTE showed multiple spindle-like extensive calcifications in myocardium (arrows). Tissue Doppler imaging revealed E’-velocity of the septal mitral annulus was 8 cm/s.

**Figure 3:**
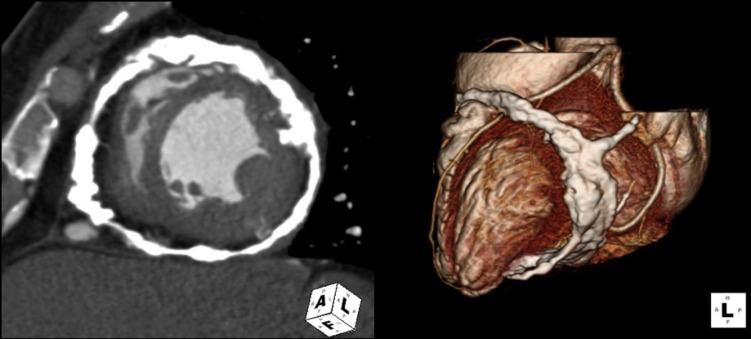
Multi-detector CT demonstrated heavy eggshell calcification encircling the heart.

A 41-year-old woman with no prior medical history visited our hospital for further evaluation of abnormal findings on two-dimensional transthoracic echocardiogram (TTE) performed during a routine health check-up examination. The patient showed mild dyspnea on ordinary physical activity with New York Heart Association class II and her physical examination was normal with a heart rate of 65 beats per minute and blood pressure of 110/60 mmHg. Initial electrocardiogram showed lower voltage complexes and chest roentgenogram revealed normal cardiac size with calcification of the pericardium (Fig. 1, arrows). On TTE, there were multiple spindle-like extensive calcifications in myocardium (Fig. 2, arrows). Left ventricle (LV) had normal chamber size (50 mm at end-diastole and 32 mm at end-systole) and wall dimensions (inter-ventricular septal wall thickness: 9 mm and LV posterior wall thickness: 9 mm) and systolic function measured as 64%. Mitral annular and aortic cuspal calcifications were not seen on TTE. Tissue Doppler imaging revealed that E’-velocity of the septal mitral annulus was 8 cm/s indicating constrictive physiology (Fig. 2). TTE did not show significant valvular regurgitations or stenoses of more than mild grade. We performed 128-slice multi-detector CT, which demonstrated heavy eggshell calcification of the pericardium encircling the heart (Fig. 3). She refused open cardiac surgery and has been followed up with outpatient clinic visits.

## Discussion

Pericardial calcification is thought to result from an inflammatory or traumatic event leads to fibro-calcific synechiae between pericardium and epicardium [1-4]. Up to two-thirds of all cases of pericardial calcification are unknown etiology [1-4]. Possible causative factors include Coxsackie B virus, radiation therapy, trauma, cardiac surgery, tuberculosis, malignancy, inflammatory and connective tissue diseases [1-4]. The presence of calcification denotes a chronic course where any causative factors induce a chronic intra-pericardial inflammation and subsequent healing with granulation tissue formation leading to development of adhesion and calcification. This in turn may cause the symptoms observed of CP [1-4]. Pericardial calcification is a common finding in patients with CP [1-3]. Careful examination of chest roentgenogram may raise the suspicion of calcific CP which showing the calcification as a mass or sheet over the heart [1, 7]. Echocardiography is a relatively simple and highly sensitive technique to differentiate between CP and restrictive cardiomyopathy. An early diastolic velocity of the lateral or septal mitral annulus of > 8 cm/s by pulse tissue Doppler is the generally accepted cut-off to differentiate CP and restrictive cardiomyopathy [1, 5]. CT scan allows a nice anatomic delineation of the pericardium and its calcification [1, 5, 6]. Furthermore, CT best defines the asymmetric degree of pericardial thickening or calcification, which may be important in determining the optimal surgical approach for pericardial resection [1, 5, 6]. The standard treatment is surgery, which is usually achieved by pericardiectomy through a median sternotomy or lateral thoracotomy [1]. Our case has some interesting features. First, multiple spindle-like extensive calcifications in myocardium in this case is a rare finding in echocardiographic examinations. Second, pericardial calcification in our case is associated with idiopathic disease and is encircling right and left ventricles despite the pericardial calcification as described elsewhere was predominantly over the right atrium and ventricle, diaphragmatic surface, and AV grooves [1, 8]. We presented an interesting case of heavy eggshell calcification of idiopathic chronic CP diagnosed by echocardiography and multi-detector CT.
